# The increased ratio of 11β-hydroxysteroid dehydrogenase type 1 versus 11β-hydroxysteroid dehydrogenase type 2 in chronic periodontitis irrespective of obesity

**DOI:** 10.1186/s40064-016-1679-6

**Published:** 2016-01-16

**Authors:** Takaya Nakata, Atsuko Fujita, Makoto Umeda, Hiroaki Yoshida, Kaoru Inami, Hiroaki Masuzaki, Hirofumi Sawai

**Affiliations:** Department of Periodontology, Osaka Dental University, 8-1 Kuzuhahanazonocho, Hirakata, Osaka 573-1121 Japan; First Department of Oral and Maxillofacial Surgery, Osaka Dental University, 8-1 Kuzuhahanazonocho, Hirakata, Osaka 573-1121 Japan; Department of Orthodontics, Osaka Dental University, 8-1 Kuzuhahanazonocho, Hirakata, Osaka 573-1121 Japan; Division of Endocrinology, Diabetes and Metabolism, Hematology, Rheumatology (Second Department of Internal Medicine), Graduate School of Medicine, University of the Ryukyus, 1 Senbaru, Nishiharacho, Nakagamigun, Okinawa 903-0213 Japan; Department of Internal Medicine, Osaka Dental University, 8-1 Kuzuhahanazonocho, Hirakata, Osaka 573-1121 Japan

**Keywords:** 11β-Hydroxysteroid dehydrogenase type 1, 11β-Hydroxysteroid dehydrogenase type 2, Chronic periodontitis, Cortisol, Obesity

## Abstract

11β-hydroxysteroid dehydrogenase type 1 (11β-HSD1), which converts inactive cortisone to active cortisol, has been reported to play an important role in metabolic diseases as well as chronic inflammatory diseases. The involvement of 11β-HSD1 in chronic periodontitis was investigated in the present study. The relationship between the levels of 11β-HSD1, chronic periodontitis, and body mass index (BMI) was analyzed. The expression of 11β-HSD1 mRNA was significantly higher in the chronic periodontitis group than in the control group. Since the expression of 11β-HSD2, which converts active cortisol to inactive cortisone, was slightly lower in the chronic periodontitis group than in the controls, the ratio of 11β-HSD1 versus 11β-HSD2 was significantly higher in the chronic periodontitis group than in the controls. A correlation was not observed between BMI and the level of 11β-HSD1 or between BMI and the ratio of 11β-HSD1 versus 11β-HSD2. These results suggested that an increase in the ratio of 11β-HSD1 versus 11β-HSD2 was associated with chronic periodontitis irrespective of obesity.

## Background

Cortisol, a major glucocorticoid hormone in humans, is known to have various effects on the metabolism of carbohydrates, fat, and proteins as well as immune responses: briefly, it promotes the conversion of proteins and lipids into glucose, and limits and resolves the inflammatory process (Rhen and Cidlowski 2005; Gross and Cidlowski 2008). It has been reported that salivary cortisol levels were positively associated with the extent and severity of chronic periodontitis (Genco et al.^1998^; Hilgert et al. 2006; Ishisaka et al. 2007; Rosania et al. 2009; Rai et al. 2011; Haririan et al. 2012; Mesa et al. 2014). With regard to serum cortisol levels, a report showed no significant differences between the chronic periodontitis patients and the controls (Mengel et al. 2002), whereas another report demonstrated that serum cortisol levels were significantly higher in patients with severe periodontitis than the controls in older Japanese adults who had never smoked (Ishisaka et al. 2008). Furthermore, it was reported that ligature-induced experimental periodontitis was more severe in Fischer 344 rats, which mount a strong hypothalamic–pituitary–adrenal (HPA) axis response leading to the increased levels of corticosterone (a major glucocorticoid in rodents), than in MHC-identical but HPA low-responding Lewis rats, suggesting the causal relationship between the increased glucocorticoid levels and severity of periodontitis (Breivik et al. 2000, 2001).

Among the many enzymes involved in the biosynthesis and catabolism of cortisol, 11β-hydroxysteroid dehydrogenase (11β-HSD) catalyzes the conversion between active cortisol and inactive cortisone, and is involved in the tissue-specific intracellular regulation of the actions of glucocorticoids; 11β-HSD type 1 (11β-HSD1) converts inactive cortisone to active cortisol, whereas 11β-HSD type 2 (11β-HSD2) catalyzes the opposite reaction (Livingstone et al. 2000; Seckl and Walker 2001; Tomlinson et al. 2004; Chapman et al. 2013). 11β-HSD1 is known to be abundantly expressed in adipose tissue and the liver (Tomlinson et al. 2004). The expression of 11β-HSD1 was previously reported to be increased in the adipocytes, especially those in visceral fat, of patients with metabolic diseases (Bujalska et al. 1997; Rask et al. 2001; Paulsen et al. 2007). Furthermore, transgenic mice selectively overexpressing 11β-HSD1 in adipose tissue showed phenotypes similar to metabolic diseases including visceral fat obesity, insulin resistance, dyslipidemia, and hypertension (Masuzaki et al. 2001, 2003). In contrast, 11β-HSD1-deficient mice were protected against metabolic diseases with overnutrition (Kotelevtsev et al. 1997). Moreover, inhibitors of 11β-HSD1 have been shown to ameliorate metabolic diseases and prevent atherosclerosis in mice (Hermanowski-Vosatka et al. 2005; Nuotio-Antar et al. 2007). These findings suggest that 11β-HSD1 plays a crucial role in metabolic diseases, to which inhibitors of 11β-HSD1 may be applied as novel therapeutics. Indeed, several highly potent and selective 11β-HSD1 inhibitors have been developed for the treatment of metabolic diseases including type 2 diabetes mellitus (Rosenstock et al. 2010; Feig et al. 2011; Anil et al. 2014; Okazaki et al. 2014; Hamilton et al. 2015).

A relationship is known to exist between metabolic diseases and chronic periodontitis (Saito et al. 1998; Shimazaki et al. 2007; D’Aiuto et al. 2008; Morita et al. 2010). Although the expression of 11β-HSD1 was recently detected in oral fibroblasts and keratinocytes (Cirillo et al. 2011), the involvement of 11β-HSD1 in chronic periodontitis has not yet been examined. We recently reported preliminary results that the expression level of 11β-HSD1 in gingival tissues from patients with chronic periodontitis was higher than that in the controls (Shiraishi et al. 2013). In the present study, we further investigated the expression of 11β-HSD1 as well as 11β-HSD2, and analyzed the relationships between the expression of 11β-HSD1/2, chronic periodontitis, and obesity.

## Methods

### Participants

Periodontal tissues were obtained from patients diagnosed with chronic periodontitis (n = 38), while those surrounding teeth extracted from patients for orthodontic treatments were used as controls (n = 14). Informed consent was obtained from all individual participants (ages between 30 and 70 years) included in this study. This study was approved by the Ethics Committee at Osaka Dental University on September 16, 2009 (approval number: 90921).

### Clinical periodontal examination

Chronic periodontitis was defined as probing depth of >3 mm, bleeding on probing, and radiographically determined alveolar bone resorption of more than one-third of the root of the tooth, whereas control group patients did not have chronic periodontitis. The dentists who performed the clinical periodontal examination were the members of the Department of Periodontology, Osaka Dental University, and therefore they used the same criteria for the diagnosis of chronic periodontitis. Periodontal tissues, including both epitheliums and connective tissues, were surgically removed when probing depth after the basic periodontal treatment (including plaque control, scaling, and root plaining) was ≥4 mm. When the tooth mobility was class III in Miller classification and alveolar bone resorption was more than two-thirds of the root of the tooth at the first examination, the tooth was extracted without the basic periodontal treatment and periodontal tissues surrounding the extracted tooth were used for experiments.

### RNA extraction

Total RNA was extracted from each sample of periodontal tissue using TRIzol Reagent (Invitrogen, Carlsbad, CA, USA) according to the manufacturer’s protocol. Briefly, each tissue sample of 50–100 mg was homogenized in 1 ml of the reagent, to which 0.2 ml chloroform was added. After centrifugation at 12,000×*g* for 15 min at 4 °C, the aqueous phase was transferred to a new tube. A total of 0.5 ml isopropyl alcohol was then added and mixed. After centrifugation at 10,000×*g* for 10 min at 4 °C, the supernatant was removed and the RNA precipitate was washed with 1 ml of 75 % alcohol and centrifuged at 7500×*g* for 5 min at 4 °C. After the supernatant was removed, the RNA pellet was air-dried and dissolved in RNase-free water. The quantity of RNA was calculated according to the absorbance at 260 nm, and the purity of RNA was assessed by the spectrometric absorbance ratio at 260 nm/280 nm.

### Real-time reverse transcription (RT)-polymerase chain reaction (PCR)

RT-PCR was performed using TaqMan^®^ RNA-to-C_T_™ 1-Step Kit and StepOnePlus™ Real Time PCR System (Applied Biosystems, Foster City, CA, USA). TaqMan^®^ Gene Expression Assays for 11β-HSD1 (#Hs01547870), 11β-HSD2 (#Hs00388669), receptor activator of nuclear factor-κB ligand (RANKL) (#Hs00243522), receptor activator of nuclear factor-κB (RANK) (#Hs00187192), osteoprotegerin (OPG) (#Hs00171068), and glyceraldehyde 3-phosphate dehydrogenase (GAPDH) (#Hs02758991) were purchased from Applied Biosystems (Foster City, CA, USA). According to the manufacturer’s protocol, 1 μl of each probe was mixed with 0.5 μl of the RT Enzyme Mix, 10 μl of the RT-PCR Mix, template (100 ng RNA), and Nuclease-free water (total volume: 20 μl). RNA was reverse transcribed at 48 °C for 15 min followed by 95 °C for 10 min. PCR was performed up to 45 cycles at 95 °C for 15 s for denaturing and 60 °C for 1 min for annealing/extension. The values of 11β-HSD1, 11β-HSD2, RANKL, RANK, and OPG mRNA relative to GAPDH mRNA were calculated in each experiment.

### Body mass index (BMI)

BMI is defined as body weight (kg) divided by the square of body height (m).

### Statistical analyses

Statistical analyses were performed using SPSS software version 21.0 (IBM, Armonk, NY, USA). The characteristics of patients with chronic periodontitis and controls were compared using the χ^2^ test for categorical variables and the *t* test for continuous variables. The Mann–Whitney *U* test was performed to compare mRNA expression levels between the control group and chronic periodontitis group or between obese and non-obese participants. Significance testing of Spearman’s correlation coefficient between BMI and the level of 11β-HSD1 or between BMI and the ratio of 11β-HSD1 versus 11β-HSD2 was also performed.

## Results

The characteristics of the study participants are summarized in Table 1. No significant differences were observed in sex, age, height, weight, or BMI between the chronic periodontitis group and control group.

**Table 1:** Characteristics of the study participants

	Control group (n = 11)	Periodontitis group (n = 38)	*P* value
No. of females/males	6/5	18/20	0.68*
Age (years)	49.1 ± 12.6	52.3 ± 11.6	0.43^†^
Height (cm)	163.5 ± 6.3	163.8 ± 9.6	0.91^†^
Weight (kg)	57.0 ± 13.0	60.4 ± 13.7	0.47^†^
BMI (kg/m^2^)	21.1 ± 3.3	22.3 ± 3.3	0.30^†^

Except where otherwise indicated, values are the mean ± SD

* χ^2^ test

^†^ *t* test

**Fig. 1:**
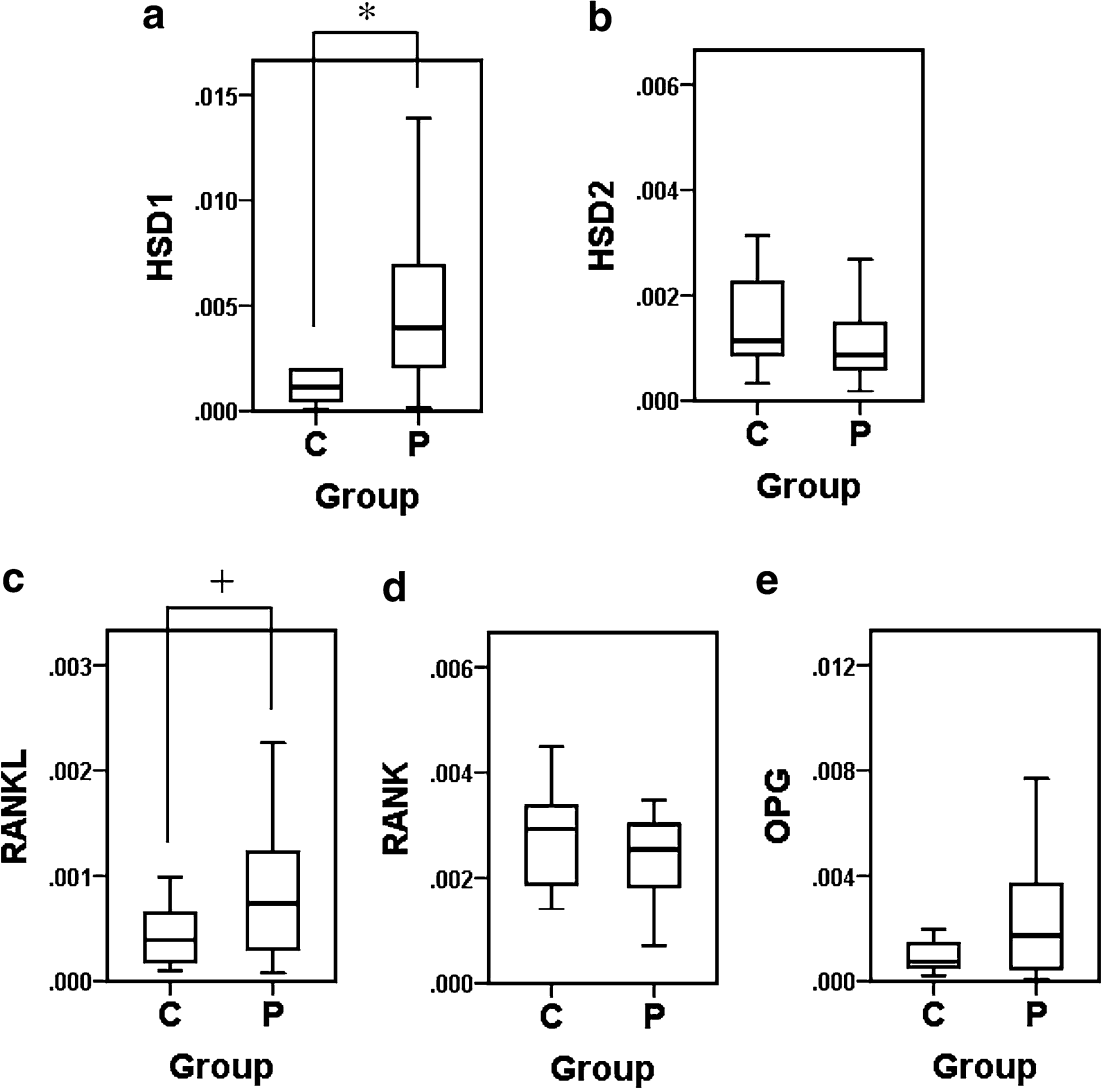
Comparison of mRNA levels in gingival tissues between the chronic periodontitis group (*P*) and control group (*C*). *Box* and *whisker plots* of the values of each mRNA relative to GAPDH mRNA are shown. Outliers are omitted. **a** 11β-HSD1; **b** 11β-HSD2; **c** RANKL; **d** RANK; **e** OPG. *P < 0.01; ^+^ *P* < 0.05

**Fig. 2:**
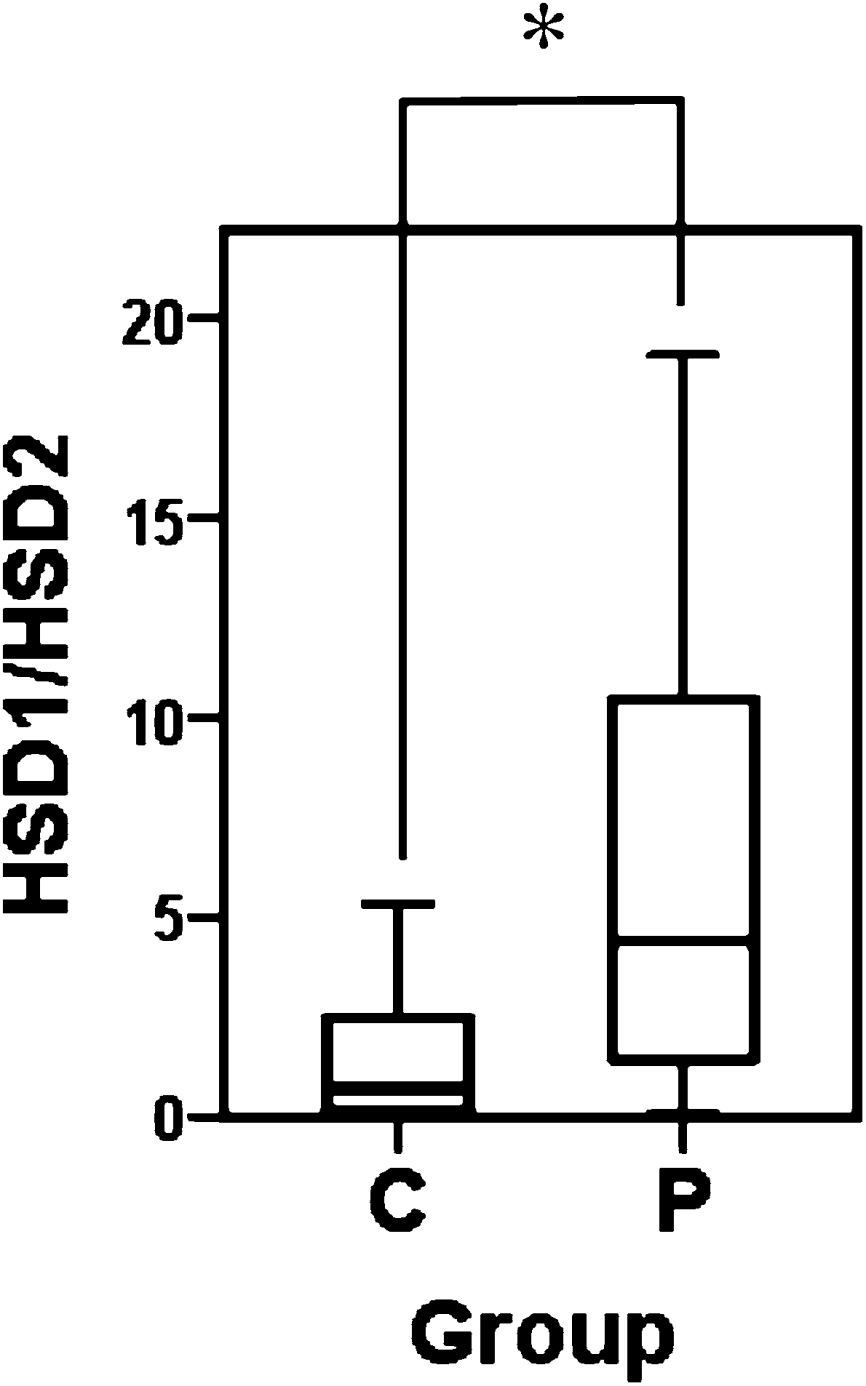
Comparison of the ratio of 11β-HSD1 versus 11β-HSD2 mRNA in gingival tissues between the chronic periodontitis group (*P*) and control group (*C*). *Box* and *whisker plots* of the ratio of 11β-HSD1 versus 11β-HSD2 mRNA are shown. Outliers are omitted. *P < 0.01

The expression levels of 11β-HSD1 and 11β-HSD2 relative to GAPDH mRNA were analyzed using the real-time RT-PCR method. The expression levels of RANKL, RANK, and OPG mRNA were also examined, since these molecules are involved in osteoclast differentiation and it was reported that the expression of RANKL was higher and that of OPG was lower in the periodontitis group than healthy control (Vernal et al. 2006; Bostanci et al. 2007; Wara-aswapati et al. 2007). As shown in Fig. 1, the values of 11β-HSD1 (Fig. 1a) and RANKL (Fig. 1c) were significantly (*P* = 0.005 and *P* = 0.014, respectively) higher in the periodontitis group than in the control group. Furthermore, the values of 11β-HSD2 (Fig. 1b) were slightly lower in the periodontitis group than in the control group. Thus, the ratio of 11β-HSD1 versus 11β-HSD2 was significantly (*P* = 0.001) higher in the periodontitis group than in the control group (Fig. 2). No significant differences were observed in the expression levels of RANK (Fig. 1d) and OPG (Fig. 1e) between the two groups.

**Fig. 3:**
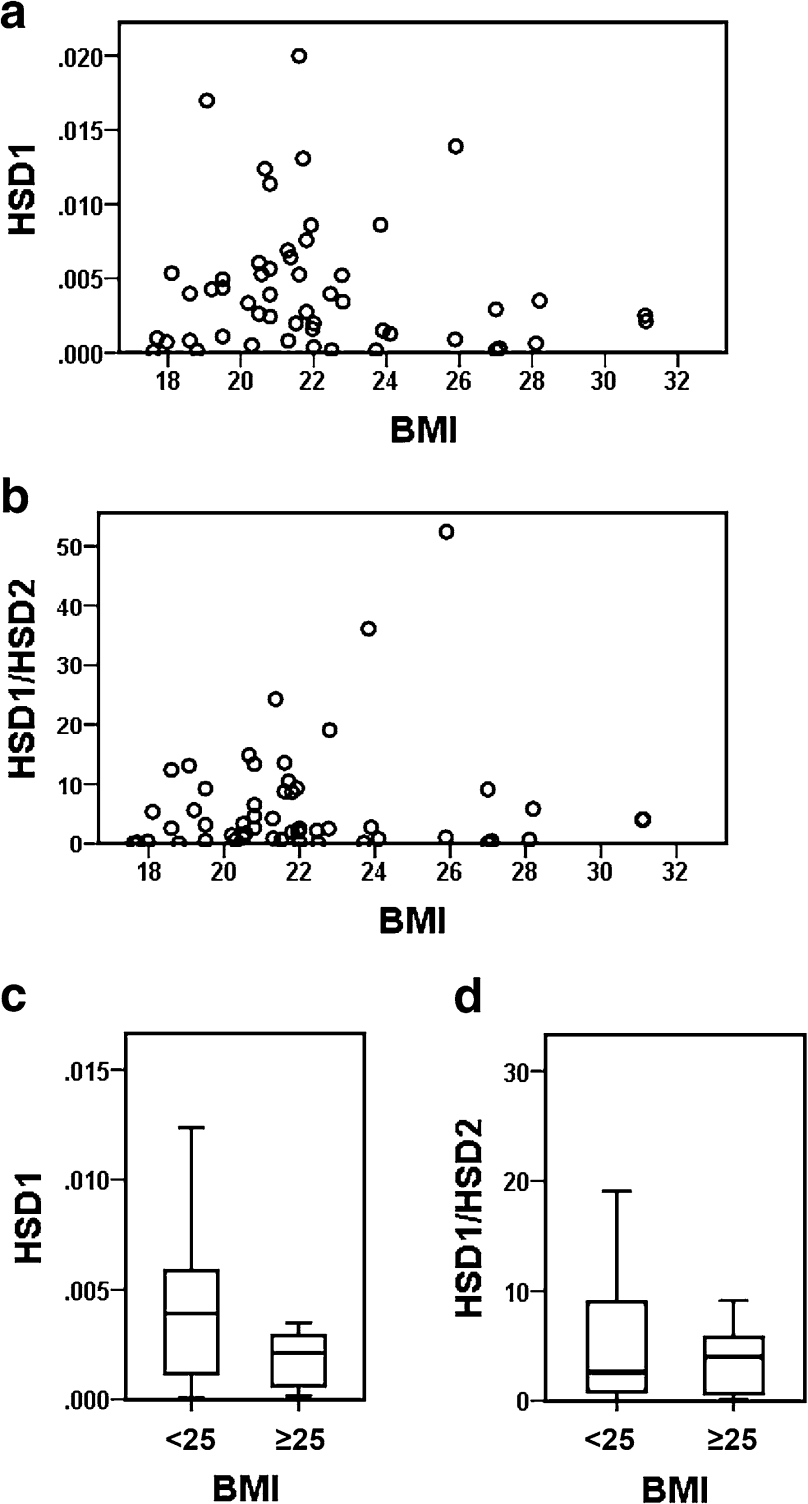
No significant correlation between body mass index (BMI) and 11β-HSD1. **a** Scatter plot of BMI versus the expression level of 11β-HSD1 mRNA. **b** Scatter plot of BMI versus the ratio of 11β-HSD1 versus 11β-HSD2 mRNA. **c** Comparison of the level of 11β-HSD1 mRNA between obese (BMI ≥ 25) and non-obese (BMI < 25) participants. *Box* and *whisker plots* of the level of 11β-HSD1 mRNA are shown. Outliers are omitted. **d** Comparison of the ratio of 11β-HSD1 versus 11β-HSD2 mRNA between obese (BMI ≥ 25) and non-obese (BMI < 25) participants. *Box* and *whisker plots* of the ratio of 11β-HSD1 versus 11β-HSD2 mRNA are shown. Outliers are omitted

Since correlations have already been reported between 11β-HSD1 expression and metabolic diseases (Bujalska et al. 1997; Rask et al. 2001; Paulsen et al. 2007) and between metabolic diseases and chronic periodontitis (Saito et al. 1998; Shimazaki et al. 2007; D’Aiuto et al. 2008; Morita et al. 2010), the relationship between the expression of 11β-HSD1 in gingival tissues and BMI as an index of obesity was analyzed in this study. Since significance testing of Spearman’s correlation coefficient between BMI and the level of 11β-HSD1 mRNA indicated *P* = 0.539 (Fig. 3a), and that between BMI and the ratio of 11β-HSD1 versus 11β-HSD2 indicated *P* = 0.656 (Fig. 3b), a correlation was not observed between BMI and the expression of 11β-HSD1 or between BMI and the ratio of 11β-HSD1 versus 11β-HSD2. Furthermore, no significant differences were observed in the level of 11β-HSD1 mRNA (Fig. 3c) or in the ratio of 11β-HSD1 versus 11β-HSD2 (Fig. 3d) between obese (BMI ≥ 25, n = 9) and non-obese (BMI < 25, n = 43) participants (*P* = 0.171 and *P* = 0.887, respectively).

## Discussion

In the present study, we investigated the relationship between 11β-HSD1/2 and chronic periodontitis, and demonstrated that the expression of 11β-HSD1 as well as the ratio of 11β-HSD1 versus 11β-HSD2 was significantly increased in chronic periodontitis. To the best of our knowledge, this is the first study to investigate the involvement of 11β-HSD1/2 in chronic periodontitis. Since it has been reported that salivary and serum cortisol levels were positively associated with the extent and severity of periodontitis (Genco et al. 1998; Hilgert et al. 2006; Ishisaka et al. 2007; Rosania et al. 2009; Rai et al. 2011; Haririan et al. 2012; Mesa et al. 2014; Mengel et al. 2002), the increased expression of 11β-HSD1 as well as the increased ratio of 11β-HSD1 versus 11β-HSD2 would result in an increase in intracellular cortisol levels, which might play a role in the pathophysiology of chronic periodontitis.

Although a correlation has already been reported between metabolic diseases and chronic periodontitis (Saito et al. 1998; Shimazaki et al. 2007; D’Aiuto et al. 2008; Morita et al. 2010), the mechanism connecting the diseases remains unclear. Although we demonstrated that the expression of 11β-HSD1 as well as the ratio of 11β-HSD1 versus 11β-HSD2 was increased in chronic periodontitis, a correlation was not observed between BMI and the expression of 11β-HSD1 or the ratio of 11β-HSD1 versus 11β-HSD2, suggesting that 11β-HSD1 might play a role in chronic periodontitis irrespective of obesity. In accordance with these results, the involvement of 11β-HSD1 in other chronic inflammatory diseases including inflammatory bowel diseases and rheumatoid arthritis has been reported (Zbankova et al. 2007; Hardy et al. 2008; Stegk et al. 2009). Thus, these results may implicate 11β-HSD1 in the pathophysiology of chronic inflammation.

Since it has been known that stress is associated with concurrent activation of the HPA axis and that there is a relationship between stress and periodontitis, the relationships between stress, cortisol and periodontitis have been investigated in several studies (Genco et al. 1998; Hilgert et al. 2006; Rosania et al. 2009; Rai et al. 2011; Haririan et al. 2012; Mesa et al. 2014; Mengel et al. 2002). Since 11β-HSD1 converts inactive cortisone to active cortisol intracellularly, the increased expression of 11β-HSD1 in chronic periodontitis seems to be independent of the HPA axis. However, further studies are required to determine the relationship between stress and the expression of 11β-HSD1 in chronic periodontitis.

The increased expression of RANKL in chronic periodontitis in the present study is consistent with previous findings (Vernal et al. 2006; Bostanci et al. 2007; Wara-aswapati et al. 2007). Although the expression of OPG was slightly increased in chronic periodontitis in the present study, it was reduced in two previous studies (Bostanci et al. 2007; Wara-aswapati et al. 2007). Thus, the role for OPG in chronic periodontitis remains to be determined.

There are limitations related to this study since it is a case–control study. Moreover, there are limitations of β error in this study due to a relatively small number of controls as well as obese participants.

## Conclusions

We demonstrated that the increased expression of 11β-HSD1 as well as the increased ratio of 11β-HSD1 versus 11β-HSD2 was associated with chronic periodontitis. Since a correlation was not observed between body mass index and 11β-HSD1 expression or 11β-HSD1/2 ratio, the increased 11β-HSD1 expression or 11β-HSD1/2 ratio might play a role in the pathophysiology of chronic periodontitis irrespective of obesity. Further investigations will be needed to elucidate the precise role of 11β-HSD1/2 in chronic periodontitis.
